# White matter microstructure is associated with functional, cognitive and emotional symptoms 12 months after mild traumatic brain injury

**DOI:** 10.1038/s41598-017-13628-1

**Published:** 2017-10-23

**Authors:** Torgeir Hellstrøm, Lars T. Westlye, Tobias Kaufmann, Nhat Trung Doan, Helene L. Søberg, Solrun Sigurdardottir, Wibeke Nordhøy, Eirik Helseth, Ole A. Andreassen, Nada Andelic

**Affiliations:** 1 Department of Physical Medicine and Rehabilitation, Oslo University Hospital, Oslo, Norway; 2 Institute of Clinical Medicine, Faculty of Medicine, University of Oslo, Oslo, Norway; 3 KG Jebsen Centre for Psychosis Research, NORMENT, Division of Mental Health and Addiction, Oslo University Hospital, Oslo, Norway & Institute for Clinical Medicine, University of Oslo, Oslo, Norway; 4 Department of Psychology, University of Oslo, Oslo, Norway; 5 Sunnaas Rehabilitation Hospital, Nesoddtangen, Norway; 6 Deptartment of Diagnostic Physics, Clinic of Radiology and Nuclear Medicine, Oslo University Hospital, Oslo, Norway; 7 Department of Neurosurgery, Oslo University Hospital, Oslo, Norway; 8 Institute of Health and Society, CHARM Research Centre for Habilitation and Rehabilitation Models & Services, Faculty of Medicine, University of Oslo, Oslo, Norway

## Abstract

Identifying patients at risk of poor outcome after mild traumatic brain injury (MTBI) is essential to aid prognostics and treatment. Diffuse axonal injury (DAI) may be the primary pathologic feature of MTBI but is normally not detectable by conventional imaging technology. This lack of sensitivity of clinical imaging techniques has impeded a pathophysiologic understanding of the long-term cognitive and emotional consequences of MTBI, which often remain unnoticed and are attributed to factors other than the injury. Diffusion tensor imaging (DTI) is sensitive to microstructural properties of brain tissue and has been suggested to be a promising candidate for the detection of DAI *in vivo*. In this study, we report strong associations between brain white matter DTI and self-reported cognitive, somatic and emotional symptoms at 12 months post-injury in 134 MTBI patients. The anatomical distribution suggested global associations, in line with the diffuse symptomatology, although the strongest effects were found in frontal regions including the genu of the corpus callosum and the forceps minor. These findings support the hypothesis that DTI may provide increased sensitivity to the diffuse pathophysiology of MTBI and suggest an important role of advanced Magnetic Resonance Imaging (MRI) in trauma care.

## Introduction

While the majority of patients with a mild traumatic brain injury (MTBI) show full remission^1^, a substantial minority experience persistent physical, emotional and cognitive problems^2,3^. This conglomerate of post-concussion symptoms, often including headache, dizziness, blurred vision, sleep disturbance, fatigue, psychological distress, and reduced memory and attention, may severely impact social functioning and work participation^4^. Identifying patients at increased risk of poor outcome is essential to aid prognostics and optimize treatment.

Since treatment and follow-up of MTBI patients is often concluded shortly after discharge from the emergency department, long-term cognitive and emotional consequences of the MTBI often remain undetected and are attributed to factors other than the injury. Although the complex and multidimensional pathophysiology is not completely understood, the penetrant and non-specific nature of the symptoms suggests close interactions between psychological and primary injury-related and secondary physiological factors. The symptoms also suggest a general and widespread involvement of brain connectivity rather than focal cortical or subcortical lesions. This hypothesis resonates well with the assumptions that the mechanic forces involved in MTBI may cause diffuse axonal injuries (DAIs), also referred to as traumatic axonal injuries (TAIs), e.g., those injuries due to traumatic shearing caused by decelerating and accelerating forces applied to the head due to a fall, traffic accident or assault^5^. Thus, DAI may be the primary pathologic feature of MTBI^6^. Unfortunately, the low sensitivity of conventional computerized tomography (CT) and MRI to detect DAI has led to a delayed understanding of the clinical MTBI symptoms, despite studies delineating trauma-related histopathology following even mild head trauma^7^.

Diffusion tensor imaging (DTI) is sensitive to the direction and magnitude of non-random water diffusion in the brain and provides a non-invasive and quantitative measurement of brain white matter microstructural properties and connectivity^8^. DTI-based indices have emerged as a sensitive index of the effects of MTBI on tissue microstructure, including axonal pathology and disruption of myelin^9^. DTI has been suggested as a promising candidate for the detection of human DAI *in vivo* ^6,9^ and cross-sectional associations between DTI abnormalities and functional outcome support a clinical significance^10–12^.

Most reports have found DTI abnormalities in fractional anisotropy (FA) and/or mean diffusivity (MD) in the acute phase (within two weeks after MTBI) compared with those in uninjured subjects, but the direction of the effects has varied across studies^9,13–18^. In contrast to a lower FA and higher MD, elevated FA and decreased MD have been reported at similar post-injury intervals in several studies^13,16^. However, in the chronic phase of MTBI, Lipton *et al*.^19^ reported decreased FA and increased MD in both the corpus callosum and subcortical white matter as well as in the internal capsules. Kraus *et al*.^20^ reported increased axial diffusivity (AD) and no differences in radial diffusivity (RD) in clinically heterogeneous chronic TBI patients and suggested that irreversible damage to myelin is less common in MTBI than in moderate and severe TBIs but that axonal damage is present in the chronic phase. In a meta-analysis, Eierud *et al*.^15^ found that FA was increased in studies that performed DTI within 2 weeks after MTBI and decreased when DTI was performed at longer post-injury intervals. The authors concluded that further studies focusing on DTI measures and the relationship with pre-injury status, mental health and neuropsychological functioning are needed to assess the efficacy of neuroimaging for clinical diagnosis and to guide the treatment strategies.

Here, we investigated white matter microstructural aberrations with DTI 12months post injury in a sample of 134 patients with MTBI. In conjunction with the literature reviewed above, the assumed injury-related mechanisms often causing DAI, and existing models suggesting a critical role of white matter microstructural characteristics in cognitive functions and mental health, we hypothesized that higher ratings of post-concussion and depressive symptoms 12 months after MTBI would be associated with lower directional coherence and higher diffusivity, manifested as lower FA and higher diffusion tensor eigenvalues. Due to the non-specific nature of the characteristic post-concussion symptoms and to increase sensitivity, our main outcome score was defined as the principal component from a principal component analysis across several clinical instruments assessing different aspects of cognitive and somatic symptoms. We hypothesized that there would be a relatively global anatomical distribution of effects in contrast to regionally specific associations but employed a methodological approach allowing for inference on a regional level to explore possible differential effects in different parts of the brain.

## Results

### Demographic and injury-related variables

Table 1 summarizes the demographics and clinical characteristics of the 134 included patients. At admission CT was negative in 76 patients and was unavailable for 5 patients, of which 4 had a negative and 1 a positive conventional MRI. Eleven patients (14%) with a negative CT displayed injury-related MRI findings (5 diffuse axonal injuries, 9 contusions and 2 subdural hemorrhages).

**Table 1:** Demographics and injury related variables of uncomplicated and complicated MTBIs.

Variables	Uncomplicated MTBI (n = 69)	Complicated MTBI (n = 65)	p-value
Age (years) Mean (SD)	40.7 (13.6)	40.4 (14.5)	0.913
Gender (n, %)			
- male	39 (57)	44 (68)	0.183
- female	30 (43)	21 (32)	
Education (n, %)			
- 0–12 years	31 (45)	32 (49)	0.131
- > 12 years	38 (55)	33 (51)	
Employment (n, %)			
- Yes	54 (80)	56(86)	0.323
- No	14 (20)	9 (14)	
Mechanism of injury (n, %)			
- Traffic accidents	35 (51)	22 (34)	0.108
- Falls	19 (27)	30 (46)	
- Violence	7 (10)	8 (12)	
- Other	8 (12)	5 (8)	
GCS score (n, %)			
-15	47 (68)	48 (74)	0.575
-14	19 (28)	14 (21)	
-13	3 (4)	3 (5)	
LOC (n, %)			
- yes ( < 5 min)	34 (49)	40 (62)	0.357
- yes (> = 5 min)	2 (3)	2 (3)	
- no	14 (20)	13 (20)	
- unknown	19 (28)	10 (15)	
PTA (n, %)			
- no amnesia	3 (4)	7 (11)	0.343
- < 1 h	51 (74)	45 (69)	
- > 1 < 24 h	0 (0)	1 (1)	
- unknown	15 (22)	12 (19)	
Length of acute hospital stay (days)			
Mean (SD)	2.0 (2.1)	2.9 (3.0)	0.039
Time to MRI-scan (days) 12 months			
Mean (SD)	487 (146)	463 (133)	0.317

p-values: T-test for continuous variables; Chi square for categorical variables.

Abbreviation: Glasgow Coma Scale (GCS), Loss of consciousness (LOC), Post traumatic amnesia (PTA).

Table 2 presents the mean and standard deviation (SD) of the Glasgow Outcome Scale Extended (GOSE), the Rivermead Post Concussion Symptoms Questionnaire (RPQ), and the Patient Health Questionnaire 9 (PHQ-9) assessed at a 12-month follow-up for the complicated and non-complicated group. The results indicate an overall good functional outcome (GOSE mean 7.25 (SD 0.82)) and low symptom burden (RPQ mean 13.04 (SD 14.0), PHQ-9 mean 6.35 (SD 5.33)). There were no significant differences found between the uncomplicated and complicated MTBI groups. No data were missing for GOSE, RPQ or PHQ-9.

**Table 2:** Self-reported outcome measures of patients with uncomplicated and complicated MTBI at 12 month.

Variables	Uncomplicated MTBI (n = 69)	Complicated MTBI (n = 65)	p-value
RPQ total Mean (SD)	13.29 (13.82)	12.78 (14.35)	0.836
RPQ somatic Mean (SD)	6.67 (7.44)	5.85 (6.97)	0.512
RPQ emotional Mean (SD)	3.23 (3.93)	3.52 (4.73)	0.698
RPQ cognitive Mean (SD)	3.39 (4.09)	3.42 (4.00)	0.973
GOSE Mean (SD)	7.25 (0.775)	7.26 (0.871)	0.915
PHQ 9 Mean (SD)	6.62 (5.19)	6.15 (5.50)	0.680

p-values: T-test for continuous variables.

Abbreviation: The Rivermead Post Concussion Symptoms Questionnaire (RPQ), Glasgow Outcome Scale Extended (GOSE), The Patient Health Questionnaire (PHQ-9).

### Associations with DTI

**Figure 1:**
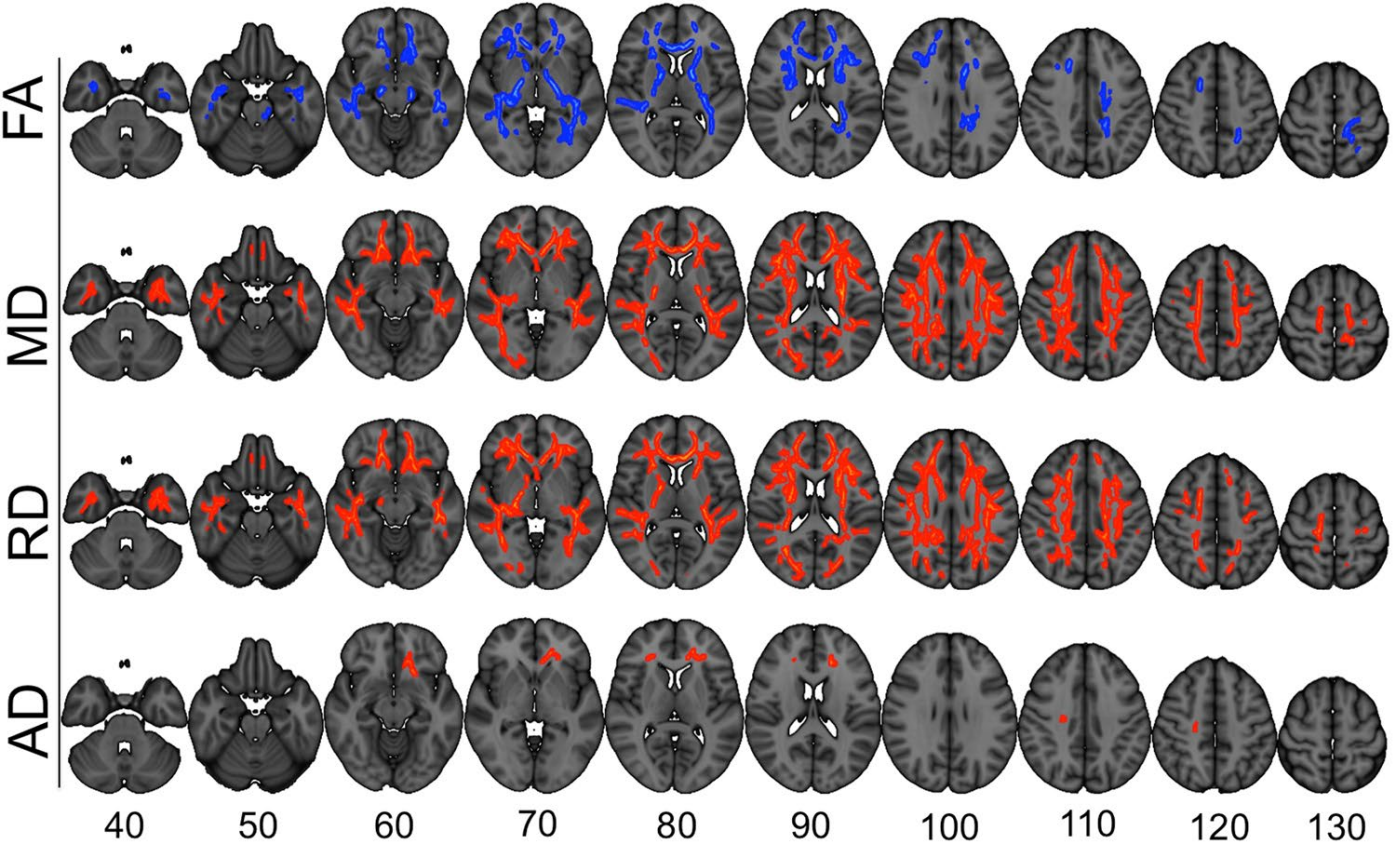
Results from the voxelwise analyses testing for associations between DTI and the composite outcome score, covarying for age, sex and head coil. Blue voxels denote higher values with better outcome and red voxels lower values with better outcome. Only voxels p < 0.05, corrected for multiple comparisons using permutation testing and TFCE are shown. Numbers reflect the z-coordinate in MNI 1 mm space.

Figure 1 shows results from the voxelwise analyses testing for associations between the composite outcome score and DTI. Permutation testing revealed significant (p < 0.05, corrected) associations for all measures, with lower FA and higher MD, RD and AD with poorer outcome. Whereas AD (t = 5.91, p < 2.81e-08, Cohen’s d = 1.04) effects were spatially confined to a small portion of the right corticospinal tract and the bilateral forceps minor and uncinate, FA (t = −6.94, p < 1.70e-10, Cohen’s d = −1.22), MD (t = 5.27, p < 5.64e-07, Cohen’s = 0.93), and RD (t = 5.45, p < 2.44e-07, Cohen’s d = 0.96) revealed a widespread pattern comprising a range of white matter pathways.

**Figure 2:**
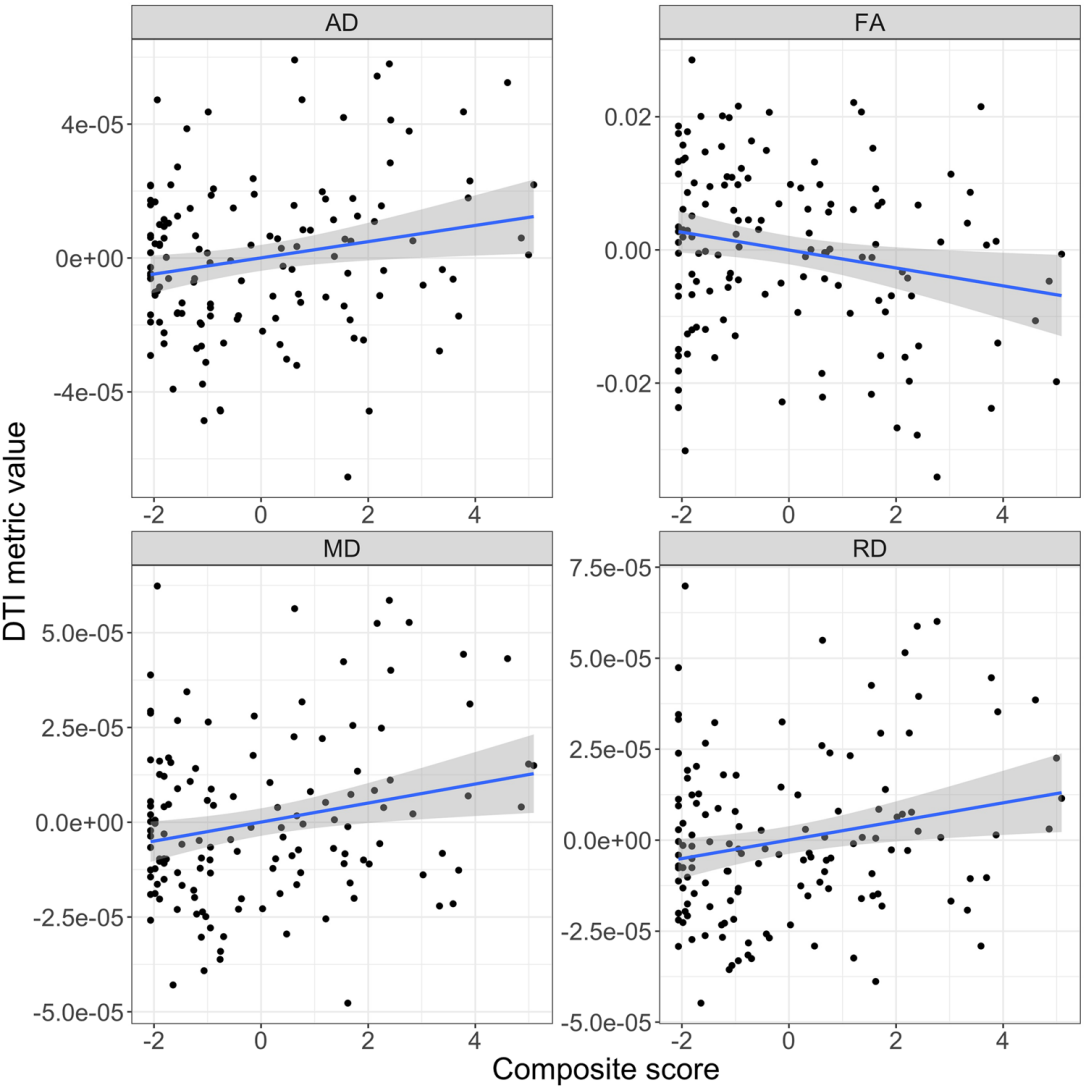
Scatter plots of the mean DTI metrics across the full TBSS skeleton versus the composite score. The effect of age, sex and head coil was regressed out before plotting and the standardized residuals of the DTI metrics are shown.

Figure 2 shows scatter plots of the mean DTI metrics across the full TBSS skeleton versus the composite score. Tables 3, 4, 5 and 6 show results from the ROI-based analysis. Briefly, linear models including age, sex and head coil as covariates revealed significant associations between the composite outcome score and mean skeleton FA (t = −2.41, p_FDR_ = 0.0173, Cohen’s d = −0.426), MD (t = 2.66, p_FDR_ = 0.00893, Cohen’s d = 0.469), RD (t = 2.59, p_FDR_ = 0.0108, Cohen’s d = 0.457), and AD (t = 2.4, p_FDR_ = 0.0177, Cohen’s d = 0.425). For atlas-based ROIs, significant associations for all DTI metrics were found for the forceps minor. The left inferior longitudinal fasciculus, bilateral inferior fronto-occipital fasciculus and the genu of the corpus callosum were significant for FA, MD and RD, and the bilateral superior longitudinal fasciculus, left superior longitudinal fasciculus (temporal part) and the right inferior longitudinal fasciculus were significant for MD, RD and AD (all p_FDR_ < 0.05). In addition, we observed strong effects of age across all DTI metrics in most ROIs and only moderate associations with sex and head coil. We revealed no major differences between the uncomplicated and complicated MTBI group except nominally significant differences with lower FA/higher RD in the body of the corpus callosum and the right uncinate in the complicated compared to the uncomplicated group. None were significant after FDR- adjustment

**Table 3:** Results from ROI-based analysis using linear models including age, sex and head coils as covariates for composite score, g.

ROI	Age						g		
	1st order polynomial term			2nd order polynomial term					
	t-value	Cohen’s d	p-value	t-value	Cohen’s d	p-value	t-value	Cohen’s d	p-value
FA - ATR L	−5.53	−0.977	1.75e-07***	−2.42	−0.428	0.017*	−2.22	−0.393	0.0279
FA - ATR R	−6.35	−1.12	3.43e-09***	−2.85	−0.504	0.00508*	−2.64	−0.467	0.00921*
FA - CG L	−7.47	−1.32	1.09e-11***	−2.13	−0.376	0.0355*	−2.24	−0.396	0.0269
FA - CG R	−4.33	−0.765	3.04e-05***	−2.35	−0.415	0.0203*	−2.24	−0.397	0.0266
FA - CING L	0.214	0.0377	0.831	−2.16	−0.382	0.0328*	−0.573	−0.101	0.567
FA - CING R	1.03	0.183	0.303	−1.55	−0.274	0.123	−0.502	−0.0887	0.617
FA - CST L	−5.9	−1.04	3.01e-08***	−2.28	−0.402	0.0245*	−2	−0.354	0.0473
FA - CST R	−4.63	−0.818	8.86e-06***	−1.81	−0.319	0.0734	−1.32	−0.233	0.189
FA - FMAJ	−5.23	−0.925	6.63e-07***	−1.6	−0.284	0.111	−1.07	−0.189	0.288
FA - FMIN	−9.97	−1.76	1.11e-17***	−1.36	−0.241	0.175	−2.89	−0.511	0.00449*
FA - IFOF L	−8.01	−1.42	6.03e-13***	−2.44	−0.431	0.0162*	−2.46	−0.435	0.0153*
FA - IFOF R	−7.46	−1.32	1.17e-11***	−2.13	−0.377	0.0349*	−2.56	−0.452	0.0117*
FA - ILF L	−7.23	−1.28	3.9e-11***	−2.81	−0.496	0.00577*	−2.59	−0.458	0.0107*
FA - ILF R	−6.12	−1.08	1.04e-08***	−3.04	−0.538	0.00286*	−1.88	−0.332	0.0627
FA - SLF L	−7.23	−1.28	3.9e-11***	−3	−0.53	0.00324*	−1.93	−0.342	0.0555
FA - SLF R	−7.47	−1.32	1.11e-11***	−2.89	−0.511	0.00449*	−1.69	−0.299	0.0935
FA - SLFT L	−4.39	−0.777	2.31e-05***	−3.43	−0.606	0.000818**	−0.728	−0.129	0.468
FA - SLFT R	−7.09	−1.25	8.21e-11***	−1.02	−0.179	0.312	−1.31	−0.232	0.193
FA - UF L	−4.93	−0.872	2.49e-06***	−1.9	−0.337	0.0591	−1.86	−0.329	0.0651
FA - UF R	−5.07	−0.897	1.33e-06***	−2.15	−0.38	0.0337*	−1.45	−0.257	0.148
FA - CC body	−5.78	−1.02	5.35e-08***	−1.86	−0.329	0.0653	−1.22	−0.215	0.225
FA - CC genu	−7.72	−1.36	2.94e-12***	0.606	0.107	0.546	−2.72	−0.482	0.00733*
FA - CC splenium	−4.98	−0.88	2.05e-06***	−0.0946	−0.0167	0.925	−0.192	−0.034	0.848
FA - ws	−7.88	−1.39	1.23e-12***	−2.75	−0.486	0.00686*	−2.41	−0.426	0.0173*
AD - ATR L	0.278	0.0491	0.782	2.82	0.498	0.00561*	0.0706	0.0125	0.944
AD - ATR R	1.28	0.226	0.204	3.32	0.587	0.00118**	−0.0228	−0.00404	0.982
AD - CG L	−5.19	−0.917	8.05e-07***	0.795	0.141	0.428	1.84	0.325	0.0681
AD - CG R	−3.19	−0.564	0.0018**	0.96	0.17	0.339	1.41	0.25	0.161
AD - CING L	−2.88	−0.51	0.0046**	1.26	0.223	0.21	1.06	0.187	0.293
AD - CING R	−3.13	−0.553	0.00217**	1.04	0.185	0.298	1.9	0.336	0.0596
AD - CST L	−8.53	−1.51	3.53e-14***	2.79	0.494	0.00601*	2.14	0.379	0.0338
AD - CST R	−7.03	−1.24	1.08e-10***	3.3	0.583	0.00127**	2.22	0.393	0.0279
AD - FMAJ	−2.17	−0.384	0.0315*	1.28	0.227	0.202	1.82	0.322	0.0708
AD - FMIN	−5.06	−0.895	1.42e-06***	3.12	0.551	0.00224*	3.15	0.557	0.00203*
AD - IFOF L	−1.88	−0.333	0.0618	1.87	0.33	0.0641	2.03	0.359	0.0443
AD - IFOF R	0.0537	0.00949	0.957	2.34	0.414	0.0208*	2.08	0.368	0.0391
AD - ILF L	−1.7	−0.301	0.0913	2.33	0.413	0.0211*	2.22	0.393	0.0281
AD - ILF R	−1.28	−0.225	0.204	1.47	0.259	0.145	2.45	0.434	0.0155*
AD - SLF L	−2.44	−0.432	0.0159*	2.43	0.43	0.0164*	2.6	0.46	0.0103*
AD - SLF R	−1.04	−0.184	0.3	2.39	0.422	0.0184*	2.74	0.484	0.00706*
AD - SLFT L	−0.491	−0.0867	0.625	2.18	0.385	0.0311*	2.57	0.453	0.0115*
AD - SLFT R	−0.789	−0.139	0.432	2.9	0.513	0.00434*	2.71	0.479	0.00771*
AD - UF L	−1.83	−0.323	0.07	2.7	0.478	0.0078*	1.95	0.345	0.0533
AD - UF R	−2.71	−0.479	0.00767*	2.15	0.379	0.0337*	1.67	0.295	0.0971
AD - CC body	−2.07	−0.366	0.0403	3.61	0.637	0.000445**	0.039	0.0069	0.969
AD - CC genu	−2.84	−0.502	0.00522**	3.28	0.579	0.00135**	1.75	0.309	0.0827
AD - CC splenium	−1.54	−0.272	0.127	2.49	0.441	0.014*	0.722	0.128	0.472
AD - ws	−3.17	−0.561	0.00188**	3.36	0.593	0.00104**	2.4	0.425	0.0177*

False discovery rate (FDR) was used to adjust the p. values for multiple comparisons. Fractional Anisotropy and Axial Diffusivity.

Abbreviations: Fractional anisotropy (FA), Axial diffusivity (AD), Anterior thalamic radiation (ATR), Corpus callosum (CC), Splenium (Splen.), Cingulum (cingulate gyrus, CG), Cingulum (hippocampus, CING), Corticospinal tract (CST), Forceps major (FMAJ), Forceps minor (FMIN), Inferior fronto-occipital fasciculus (IFOF), Inferior longitudinal fasciculus (ILF), Superior longitudinal fasciculus (SLF), Superior longitudinal fasciculus (temporal part, SLFT), Uncinate fasciculus (UF), Whole Skeleton (WS).

**Table 4:** Results from ROI-based analysis using linear models including age, sex and head coils as covariates for composite score, g.

ROI	Age						g		
	1st order polynomial term			2nd order polynomial term					
	t-value	Cohen’s d	p-value	t-value	Cohen’s d	p-value	t-value	Cohen’s d	p-value
MD - ATR L	3.03	0.535	0.00298**	2.84	0.503	0.0052*	0.589	0.104	0.557
MD - ATR R	4.09	0.722	7.68e-05***	3.5	0.619	0.000633**	0.635	0.112	0.526
MD - CG L	1.74	0.307	0.0849	2.48	0.438	0.0145*	3.26	0.577	0.00142*
MD - CG R	1.05	0.186	0.295	2.89	0.511	0.0045*	3.09	0.547	0.00244*
MD - CING L	−2.04	−0.36	0.0435	2.15	0.38	0.0336*	1.09	0.192	0.279
MD - CING R	−2.84	−0.503	0.00518**	1.76	0.312	0.0804	1.57	0.278	0.119
MD - CST L	−2.24	−0.396	0.0269*	2.88	0.508	0.00473*	2.51	0.444	0.0133*
MD - CST R	−2.28	−0.403	0.0241*	2.97	0.525	0.00353*	2.21	0.39	0.029
MD - FMAJ	1.77	0.313	0.0789	1.86	0.329	0.0654	1.7	0.3	0.0922
MD - FMIN	1.29	0.227	0.2	2.77	0.49	0.00638*	3.84	0.679	0.000193*
MD- IFOF L	3.08	0.544	0.00256**	2.71	0.479	0.00766*	2.82	0.498	0.00559*
MD - IFOF R	4.06	0.718	8.39e-05***	2.9	0.513	0.00433*	2.92	0.517	0.00409*
MD - ILF L	2.42	0.428	0.0169*	3.08	0.544	0.00254*	2.97	0.525	0.00354*
MD - ILF R	1.94	0.343	0.0546	2.59	0.458	0.0107*	2.72	0.481	0.00742*
MD - SLF L	2.42	0.428	0.0169*	3.27	0.578	0.00139**	2.67	0.471	0.00868*
MD - SLF R	3.14	0.555	0.00212**	3.21	0.567	0.00168**	2.67	0.473	0.00848*
MD - SLFT L	2.24	0.396	0.0267*	3.38	0.598	0.000956**	2.4	0.424	0.018*
MD - SLFT R	2.74	0.484	0.0071*	2.95	0.521	0.00381*	2.88	0.509	0.00465*
MD - UF L	1.28	0.226	0.204	3.18	0.562	0.00186**	2.54	0.449	0.0123*
MD - UF R	0.473	0.0837	0.637	2.77	0.49	0.00643*	2.07	0.366	0.0407
MD - CC body	3.5	0.618	0.000645**	3.69	0.652	0.00033**	0.671	0.119	0.503
MD - CC genu	3.45	0.61	0.000764**	2.18	0.386	0.0309*	3.23	0.57	0.00159*
MD - CCsplenium	1.93	0.341	0.0558	2.14	0.379	0.034*	0.656	0.116	0.513
MD - ws	2.34	0.413	0.021*	3.52	0.623	0.00059**	2.66	0.469	0.00893*
RD - ATR L	4.54	0.802	1.3e-05***	2.72	0.481	0.00736*	0.872	0.154	0.385
RD - ATR R	5.59	0.988	1.33e-07***	3.43	0.607	0.000807**	1	0.177	0.318
RD - CG L	5.14	0.908	1.01e-06***	2.71	0.479	0.00766*	3.12	0.551	0.00225*
RD - CG R	3.44	0.607	0.000797**	3.1	0.547	0.00242*	3.06	0.541	0.00271*
RD - CING L	−1.13	−0.199	0.262	2.41	0.426	0.0173*	0.932	0.165	0.353
RD - CING R	−2.25	−0.398	0.0259*	2.01	0.356	0.0461	1.12	0.199	0.263
RD - CST L	1.59	0.281	0.114	2.61	0.462	0.0101*	2.45	0.433	0.0157*
RD - CST R	0.834	0.147	0.406	2.44	0.431	0.016*	1.95	0.345	0.0534
RD - FMAJ	3.49	0.618	0.000654**	1.86	0.329	0.0649	1.37	0.242	0.173
RD - FMIN	5.2	0.919	7.7e-07***	2.13	0.376	0.0352*	3.7	0.655	0.000316*
RD - IFOF L	5.6	0.989	1.27e-07***	2.83	0.5	0.0054*	2.89	0.511	0.0045*
RD - IFOF R	5.86	1.04	3.64e-08***	2.85	0.503	0.00514*	3.02	0.535	0.00301*
RD - ILF L	4.55	0.805	1.22e-05***	3.19	0.563	0.00182**	3.09	0.547	0.00244*
RD - ILF R	3.63	0.641	0.000412***	3	0.53	0.00328*	2.62	0.462	0.00999*
RD - SLF L	4.82	0.852	3.99e-06***	3.43	0.606	0.000812**	2.46	0.435	0.0151*
RD - SLF R	5.17	0.913	8.91e-07***	3.38	0.598	0.000959**	2.4	0.425	0.0176*
RD - SLFT L	3.59	0.635	0.000462**	3.6	0.636	0.000456**	1.91	0.338	0.0581
RD - SLFT R	4.86	0.86	3.32e-06***	2.48	0.439	0.0144*	2.52	0.446	0.0129*
RD - UF L	3.13	0.553	0.00217**	3	0.53	0.00326*	2.53	0.448	0.0125*
RD - UF R	2.67	0.472	0.0086*	2.79	0.494	0.00601*	2.04	0.36	0.0439
RD - CC body	5.11	0.903	1.15e-06***	2.78	0.492	0.0062*	0.784	0.139	0.435
RD - CC genu	6.86	1.21	2.7e-10***	0.503	0.089	0.616	3.08	0.545	0.00251*
RD - CC splenium	4.09	0.723	7.57e-05***	1.2	0.212	0.232	0.401	0.0709	0.689
RD - ws	4.98	0.88	2.03e-06***	3.35	0.592	0.00108**	2.59	0.457	0.0108*

False discovery rate (FDR) was used to adjust the p. values for multiple comparisons. Mean Diffusivity and Radial Diffusivity.

Abbreviations: Mean diffusivity (MD), Radial diffusivity (RD), Anterior thalamic radiation (ATR), Corpus callosum (CC), Splenium (Splen.), Cingulum (cingulate gyrus, CG), Cingulum (hippocampus, CING), Corticospinal tract (CST), Forceps major (FMAJ), Forceps minor (FMIN), Inferior fronto-occipital fasciculus (IFOF), Inferior longitudinal fasciculus (ILF), Superior longitudinal fasciculus (SLF), Superior longitudinal fasciculus (temporal part, SLFT), Uncinate fasciculus (UF), Whole Skeleton (WS).

**Table 5:** Results from ROI-based analysis using linear models including age, sex and head coils as covariates for complicated vs. uncomplicated MTBI.

ROI	Age						Complicated vs. Uncomplicated		
	1st order polynomial term			2nd order polynomial term					
	t-value	Cohen’s d	p-value	t-value	Cohen’s d	p-value	t-value	Cohen’s d	p-value
FA - ATR L	−5.2	−0.92	7.57e-07***	−2.2	−0.389	0.0297*	−0.98	−0.173	0.329
FA - ATR R	−5.94	−1.05	2.56e-08***	−2.56	−0.453	0.0115*	−1.32	−0.234	0.188
FA - CG L	−7.19	−1.27	4.9e-11***	−1.86	−0.329	0.0652	−1.86	−0.33	0.0646
FA - CG R	−4.02	−0.711	9.76e-05***	−2.09	−0.37	0.0383	−1.61	−0.285	0.11
FA - CING L	0.312	0.0552	0.755	−2.02	−0.356	0.046	−1.73	−0.307	0.0853
FA - CING R	1.12	0.198	0.264	−1.45	−0.256	0.149	−1.03	−0.182	0.305
FA - CST L	−5.62	−0.993	1.16e-07***	−2.09	−0.369	0.0386	−0.766	−0.135	0.445
FA - CST R	−4.5	−0.796	1.49e-05***	−1.61	−0.285	0.11	−1.79	−0.316	0.0765
FA - FMAJ	−5.12	−0.906	1.08e-06***	−1.48	−0.261	0.142	−0.926	−0.164	0.356
FA - FMIN	−9.5	−1.68	1.63e-16***	−1.06	−0.187	0.293	−1.91	−0.337	0.0585
FA - IFOF L	−7.62	−1.35	5.03e-12***	−2.19	−0.386	0.0307*	−1.16	−0.205	0.247
FA - IFOF R	−7.07	−1.25	8.73e-11***	−1.85	−0.327	0.0663	−1.64	−0.289	0.104
FA - ILF L	−6.79	−1.2	3.74e-10***	−2.57	−0.455	0.0112*	−0.56	−0.0989	0.577
FA - ILF R	−5.88	−1.04	3.29e-08***	−2.82	−0.499	0.00554*	−1.39	−0.246	0.167
FA - SLF L	−6.95	−1.23	1.7e-10***	−2.86	−0.506	0.00495*	−0.0277	−0.00489	0.978
FA - SLF R	−7.25	−1.28	3.56e-11***	−2.72	−0.48	0.00752*	−0.903	−0.16	0.368
FA - SLFT L	−4.33	−0.765	3.01e-05***	−3.37	−0.597	0.000978*	−0.072	−0.0127	0.943
FA - SLFT R	−6.96	−1.23	1.6e-10***	−0.854	−0.151	0.395	−1.26	−0.222	0.212
FA - UF L	−4.71	−0.832	6.45e-06***	−1.68	−0.297	0.0959	−1.71	−0.302	0.0902
FA - UF R	−4.99	−0.882	1.96e-06***	−1.91	−0.338	0.0584	−2.62	−0.463	0.00994
FA - CC body	−5.78	−1.02	5.32e-08***	−1.62	−0.287	0.107	−2.89	−0.511	0.00453
FA - CC genu	−7.35	−1.3	2.07e-11***	0.905	0.16	0.367	−2.29	−0.405	0.0236
FA - CC splenium	−5.04	−0.89	1.57e-06***	0.0678	0.012	0.946	−2	−0.354	0.0475
FA - ws	−7.53	−1.33	8.13e-12***	−2.47	−0.437	0.0147*	−1.6	−0.283	0.112
AD - ATR L	0.263	0.0465	0.793	2.77	0.489	0.00649*	0.583	0.103	0.561
AD - ATR R	1.28	0.226	0.203	3.21	0.567	0.00169*	2.06	0.364	0.0415
AD - CG L	−5.39	−0.952	3.34e-07***	0.772	0.137	0.441	−0.944	−0.167	0.347
AD - CG R	−3.36	−0.593	0.00104**	0.9	0.159	0.37	−0.198	−0.035	0.843
AD - CING L	−3.02	−0.534	0.00304**	1.3	0.23	0.195	−1.21	−0.214	0.228
AD - CING R	−3.36	−0.594	0.00102**	0.869	0.154	0.386	0.944	0.167	0.347
AD - CST L	−8.72	−1.54	1.27e−14***	2.6	0.46	0.0104*	0.532	0.094	0.596
AD - CST R	−7.28	−1.29	2.98e-11***	3.05	0.539	0.0028*	1.26	0.223	0.209
AD - FMAJ	−2.38	−0.421	0.0187*	1.17	0.207	0.243	0.0788	0.0139	0.937
AD - FMIN	−5.29	−0.935	5.1e-07***	2.82	0.499	0.00555*	0.478	0.0845	0.633
AD - IFOF L	−2.11	−0.373	0.037	1.76	0.312	0.08	−0.313	−0.0554	0.755
AD - IFOF R	−0.201	−0.0356	0.841	2.18	0.385	0.0314*	0.34	0.06	0.735
AD - ILF L	−1.94	−0.343	0.0546	2.26	0.399	0.0257*	−0.869	−0.154	0.386
AD - ILF R	−1.54	−0.272	0.126	1.37	0.242	0.174	−0.624	−0.11	0.534
AD - SLF L	−2.7	−0.478	0.00778*	2.27	0.402	0.0247*	−0.345	−0.061	0.731
AD - SLF R	−1.35	−0.238	0.18	2.17	0.383	0.0322*	0.341	0.0604	0.733
AD - SLFT L	−0.785	−0.139	0.434	2.01	0.355	0.0466	−0.0811	−0.0143	0.936
AD - SLFT R	−1.09	−0.192	0.279	2.73	0.483	0.00716*	−0.47	−0.0831	0.639
AD - UF L	−2.05	−0.362	0.0424	2.56	0.452	0.0117*	0.139	0.0246	0.89
AD - UF R	−2.9	−0.513	0.00435**	2.03	0.359	0.0444	0.15	0.0266	0.881
AD - CC body	−2.1	−0.371	0.0378	3.57	0.631	0.000507*	0.406	0.0718	0.685
AD - CC genu	−3.04	−0.537	0.00288**	3.16	0.558	0.00198*	−0.105	−0.0186	0.916
AD - CC splenium	−1.65	−0.292	0.101	2.38	0.421	0.0187*	1.01	0.178	0.315
AD - ws	−3.43	−0.606	0.00082**	3.12	0.552	0.00221*	0.61	0.108	0.543

False discovery rate (FDR) was used to adjust the p. values for multiple comparisons. Fractional Anisotropy and Axial Diffusivity.

Abbreviations: Fractional anisotropy (FA), Axial diffusivity (AD), Anterior thalamic radiation (ATR), Corpus callosum (CC), Splenium (Splen.), Cingulum (cingulate gyrus, CG), Cingulum (hippocampus, CING), Corticospinal tract (CST), Forceps major (FMAJ), Forceps minor (FMIN), Inferior fronto-occipital fasciculus (IFOF), Inferior longitudinal fasciculus (ILF), Superior longitudinal fasciculus (SLF), Superior longitudinal fasciculus (temporal part, SLFT), Uncinate fasciculus (UF), Whole Skeleton (WS).

**Table 6:** Results from ROI-based analysis using linear models including age, sex and head coils as covariates for complicated vs. uncomplicated MTBI.

ROI	Age						Complicated vs. Uncomplicated		
	1st order polynomial term			2nd order polynomial term					
	t-value	Cohen’s d	p-value	t-value	Cohen’s d	p-value	t-value	Cohen’s d	p-value
MD - ATR L	2.97	0.525	0.00354**	2.74	0.485	0.00698*	0.965	0.171	0.336
MD - ATR R	4.09	0.723	7.57e-05***	3.35	0.593	0.00105*	2.43	0.429	0.0166
MD - CG L	1.29	0.228	0.199	2.17	0.384	0.0317*	0.82	0.145	0.414
MD - CG R	0.644	0.114	0.521	2.56	0.453	0.0116*	1.27	0.225	0.206
MD - CING L	−2.18	−0.385	0.0313*	2.08	0.368	0.0395	0.0394	0.00696	0.969
MD - CING R	−3.08	−0.544	0.00254**	1.56	0.276	0.12	1.57	0.278	0.118
MD - CST L	−2.52	−0.446	0.0129*	2.62	0.464	0.00979*	0.942	0.167	0.348
MD - CST R	−2.58	−0.457	0.0109*	2.7	0.477	0.00786*	1.93	0.341	0.0556
MD - FMAJ	1.55	0.275	0.123	1.71	0.302	0.0902	0.659	0.116	0.511
MD - FMIN	0.771	0.136	0.442	2.36	0.417	0.02*	1.46	0.258	0.146
MD - IFOF L	2.67	0.472	0.00859*	2.46	0.435	0.0153*	0.492	0.0869	0.624
MD - IFOF R	3.62	0.64	0.000422***	2.6	0.459	0.0105*	1.14	0.202	0.256
MD - ILF L	2.01	0.356	0.0463	2.85	0.505	0.00503*	−0.225	−0.0398	0.822
MD - ILF R	1.58	0.279	0.117	2.38	0.42	0.0189*	0.152	0.0269	0.879
MD - SLF L	2.06	0.364	0.0414	3.07	0.542	0.00264*	−0.167	−0.0296	0.867
MD - SLF R	2.75	0.487	0.00675*	2.95	0.521	0.00382*	0.692	0.122	0.49
MD - SLFT L	1.93	0.341	0.056	3.22	0.568	0.00165*	−0.304	−0.0538	0.761
MD - SLFT R	2.33	0.411	0.0215*	2.69	0.475	0.00815*	0.426	0.0753	0.671
MD - UF L	0.943	0.167	0.348	2.9	0.513	0.0044*	1.24	0.22	0.215
MD - UF R	0.203	0.0359	0.839	2.54	0.448	0.0124*	1.41	0.249	0.162
MD - CC body	3.49	0.616	0.000674**	3.54	0.626	0.000558*	2.53	0.448	0.0125
MD - CC genu	2.97	0.525	0.00358**	1.85	0.327	0.0668	1.5	0.266	0.136
MD –CCsplenium	1.86	0.329	0.0651	1.99	0.351	0.049	1.89	0.334	0.0613
MD - ws	1.97	0.349	0.0507	3.22	0.569	0.00163*	1.42	0.252	0.157
RD - ATR L	4.46	0.788	1.79e-05***	2.59	0.459	0.0106*	1.15	0.203	0.253
RD - ATR R	5.58	0.987	1.36e-07***	3.25	0.575	0.00146*	2.52	0.445	0.0131
RD - CG L	4.65	0.821	8.27e-06***	2.37	0.418	0.0194*	1.58	0.279	0.117
RD - CG R	2.99	0.529	0.00332**	2.74	0.484	0.00701*	1.77	0.314	0.0784
RD - CING L	−1.26	−0.223	0.21	2.29	0.406	0.0234*	0.892	0.158	0.374
RD - CING R	−2.45	−0.433	0.0156*	1.83	0.324	0.069	1.8	0.318	0.0748
RD - CST L	1.27	0.224	0.208	2.36	0.418	0.0197*	1.08	0.191	0.283
RD - CST R	0.575	0.102	0.567	2.19	0.386	0.0306*	2.12	0.376	0.0356
RD - FMAJ	3.33	0.588	0.00115**	1.72	0.304	0.0879	0.855	0.151	0.394
RD - FMIN	4.59	0.811	1.06e-05***	1.73	0.306	0.0856	1.9	0.335	0.0601
RD - IFOF L	5.12	0.906	1.07e-06***	2.54	0.45	0.0122*	0.9	0.159	0.37
RD - IFOF R	5.37	0.95	3.53e-07***	2.52	0.445	0.0131*	1.46	0.258	0.146
RD - ILF L	4.06	0.717	8.57e-05***	2.91	0.515	0.00422*	0.163	0.0287	0.871
RD - ILF R	3.25	0.574	0.00149**	2.75	0.487	0.00678*	0.589	0.104	0.557
RD - SLF L	4.45	0.787	1.83e-05***	3.24	0.572	0.00154*	−0.0579	−0.0102	0.954
RD - SLF R	4.81	0.849	4.25e-06***	3.13	0.554	0.00214*	0.828	0.146	0.409
RD - SLFT L	3.35	0.592	0.00107**	3.49	0.616	0.000673*	−0.399	−0.0705	0.691
RD - SLFT R	4.49	0.793	1.6e-05***	2.23	0.394	0.0274*	1.02	0.181	0.309
RD - UF L	2.78	0.492	0.00617*	2.7	0.477	0.00787*	1.79	0.317	0.0752
RD - UF R	2.41	0.427	0.0172*	2.53	0.448	0.0125*	2.11	0.373	0.0369
RD - CC body	5.16	0.911	9.35e-07***	2.6	0.46	0.0104*	2.86	0.505	0.00502
RD - CC genu	6.38	1.13	2.91e-09***	0.184	0.0325	0.854	2.15	0.381	0.033
RD - CC splenium	4.1	0.725	7.2e-05***	1.04	0.185	0.298	2	0.354	0.0475
RD - ws	4.61	0.815	9.57e-06***	3.04	0.537	0.00288*	1.74	0.308	0.084

False discovery rate (FDR) was used to adjust the p. values for multiple comparisons. Mean diffusivity and Radial Diffusivity.

Abbreviations: Mean diffusivity (MD), Radial diffusivity (RD), Anterior thalamic radiation (ATR), Corpus callosum (CC), Splenium (Splen.), Cingulum (cingulate gyrus, CG), Cingulum (hippocampus, CING), Corticospinal tract (CST), Forceps major (FMAJ), Forceps minor (FMIN), Inferior fronto-occipital fasciculus (IFOF), Inferior longitudinal fasciculus (ILF), Superior longitudinal fasciculus (SLF), Superior longitudinal fasciculus (temporal part, SLFT), Uncinate fasciculus (UF), Whole Skeleton (WS).

Including QA metrics (MAXVOX and tSNR, see Methods) in the models did not change the results. tSNR showed significant association with FA (t = 3.34, p_FDR_ = 0.00108, Cohen’s d = 0.591) and RD (t = −3.37, p_FDR_ = 0.00099, Cohen’s d = −0.596) in the body and RD (t = −3.29, p_FDR_ = 0.00129, Cohen’s d = −0.582) in the splenium of the corpus callosum.

## Discussion

Non-specific cognitive, somatic and emotional symptoms following MTBI often remain undetected. Conventional neuroimaging-based assessments lack the sensitivity necessary to detect diffuse axonal injuries, which represent one major potential mechanism, and the symptoms characteristically emerge long after the conclusion of the initial workup at the emergency department. Such delayed and “hidden” symptoms may therefore often be attributed to factors other than the brain injury. Here, we demonstrate robust associations between a composite measure of self-reported outcome (cognitive, somatic and emotional symptoms 12 months after MTBI) and WM microstructural properties as measured using DTI in uncomplicated and complicated MTBI. In particular, patients with a higher symptom burden showed anatomically widespread reduced directional coherence and an increased magnitude of diffusion across the primary axis of the diffusion tensor, as indexed by decreased FA and increased RD, respectively. Whereas the anatomical distribution suggested primarily global associations, in line with the diffuse symptomatology, the strongest effects were found in frontal regions, including the genu of the corpus callosum and the forceps minor. Although the lack of longitudinal DTI data precludes interpretations regarding the temporal evolution from the time of injury, our results suggest a link between brain white matter microstructure and a composite measure of self-reported outcome 12 months after the injury.

Previous studies have demonstrated an important role of DTI in evaluating white matter integrity after MTBI^21^, typically converging on lower FA for MTBI patients than for healthy controls^11,19,22,23^. The anatomical distribution of DTI abnormalities in our study, including the FA effects, are in line with the previous studies^11,19,24^. The gray-white matter junction and midline brain structures are particularly vulnerable to DAI, and the corpus callosum and dorsolateral midbrain are frequently involved because of their susceptibility to biomechanical shear-strain forces^25^. The localized AD effects found in this study are in line with a previous study that investigated patients in the post-acute/chronic TBI phase and found an overall increase in AD that was positively correlated with time since TBI and greater in the “mild” TBI group than in the controls^24^. The authors indicated that increased AD may reflect adaptive axonal recovery; however, the pathological significance of these effects remains unclear and should be assessed in relevant animal models^24^.

Studies on MTBI have correlated post-concussion cognitive dysfunction with focal white matter abnormalities^12,24,26,27^. However, few studies have attempted to correlate DTI abnormalities with patient self-reported symptoms^28^. In a recent TRACK TBI study^17^, a subgroup of 32 complicated MTBIs showed reduced FA in multiple tracts, whereas the uncomplicated MTBIs demonstrated no differences in DTI parameters compared to those in healthy controls in the subacute phase (mean, 11.2 days after injury). The regions with reduced FA were significantly associated with functional outcome 3 and 6 months post injury. Reduced FA may occur due to axonal injury or disruption of the myelin sheath^29^. In line with *Messe et al*.^18^ who found that only those MTBI patients with residual behavioral and cognitive complaints demonstrated alterations in white matter integrity compared to the white matter in healthy controls, we found an association between a comprehensive composite score of persistent symptoms and DTI. We included both asymptomatic and symptomatic individuals with both uncomplicated and complicated MTBI. Contrary to studies demonstrating lower FA with greater injury severity^30^, we found no significant differences between uncomplicated and complicated MTBI. The lack of a significant group difference may be explained by the fact that all patients in the current study needed hospitalization in a level I trauma center and thus represented a relatively homogenous population at the more severe end of the MTBI spectrum. This intepretation is in line with Kinnunen *et al*.^24^, who reported significant white matter abnormalities in patients without microbleeds compared with age-matched controls, and indicates that further refinement of the diagnostic classification of MTBI is needed.

Despite the sensitivity of DTI and its potential to identify patients at risk for poor long-term outcomes, to date, DTI has not yielded a validated prognostic biomarker for several reasons: a lack of longitudinal studies assessing the relationship between imaging and outcome^22,31^, differing intervals between injury and MRI assessment in different studies, inconsistencies in reported outcome, methodological heterogeneity, and small sample sizes.

Mental health, including levels of depression, anxiety and possible traumatic stress, has been associated with functional outcome after MTBI in several studies^32–37^. In a recent study, Spitz *et al*. found mood disorders following TBI to be associated with reduced white matter integrity in multiple fiber bundles connecting temporal and frontal brain regions^38^. Our composite score included emotional symptoms and depression, reflecting the fact that mental health factors have an important role in MTBI outcome. Various outcome measures are used for MTBI. The GOSE is often used for global outcome and RPQ for self-reported symptoms. The coarseness of GOSE makes it less sensitive to subtle dysfunction, which is typically observed in patients with MTBI, and may therefore not permit sufficient differentiation of the outcome in patients with milder injuries^39^. RPQ is sensitive to post-concussion symptoms but is a gross outcome measure and grades common symptoms that are not specific to MTBI. Because composite outcome measures that include several dimensions of functioning are likely to increase sensitivity and are in line with a recent recommendation^39^, we created a composite outcome score using a principal component analysis (PCA) based on global functioning and self-reported cognitive, emotional, somatic and mental health symptoms (GOSE, RPQ and PHQ-9, respectively). Further studies are needed to assess the clinical specificity of the DTI associations and to explore the interactions between predisposing and injury-related factors.

Finally, in line with previous large-scale cross-sectional and longitudinal studies^40,41^, we observed strong effects of age across all DTI metrics in most ROIs and only moderate associations with sex. Although further research is needed to explore this hypothesis, normal age-related effects on the brain white matter integrity may leave the older brain more vulnerable to subtle injury-related processes, therefore rendering age an important prognostic factor^42,43^.

The primary limitations of the current study include the cross-sectional design. Longitudinal studies are needed to determine the time course of DTI abnormalities, how they may change over time and how changes in the DTI indices are related to recovery of post-concussion symptoms. Because of the lack of a control group either without injury or with non-head injury, we were unable to determine whether the results are specific to MTBI. This is particularly pertinent given the demonstrated associations between normal variability in personality and mental health-related symptoms and DTI parameters even in presumably healthy individuals^44,45^. A potential caveat is that only MTBI patients requiring neurosurgical consultation at the emergency department and hospitalization were included in the present study and may therefore be in the more severe part of the mild injuries despite normal CT and MRI scan. Our results may, therefore, have the most value for more severely injured MTBI patients, and generalization of the findings to all MTBI patients should be made with caution. In addition, of the 153 patients with available DTI, 19 were discarded due to poor DTI quality, yielding a total sample of 134 patients. In our experience, excluding approximately 12% (19/153) of the available datasets due to poor quality either due to technical issues or subject motion is quite typical, in particular in clinical studies. Although excluding data based on poor data quality is inevitable, it may induce bias since subject motion and possibly other sources of noise tend to increase with increasing disease severity.

Although the 12-month interval between the incident and the follow-up assessment is in line with previous recommendations^46^, further studies with more frequent follow-ups, both in the short- and long-term, are needed to probe the temporal dynamics of the associations from the time of injury.

Indeed, the injury-to-MRI interval may be a critical factor in MTBI studies. Avariety of different biological processes within injured white matter have been postulated to vary not only according to injury severity but also at different time intervals after injury^47^. Patients in the current study underwent MRI at approximately 12 months after injury; thus, we can be more assured that the abnormalities in the present study represent a more chronic MTBI pathology. Importantly, in line with most studies on TBI, we cannot rule out that the reported results partly reflect associations that are not related to the injury but rather reflect predisposing associations such as depression or anxiety.

Associations between imaging markers and clinical phenotypes may be confounded by data quality, e.g., due to increases in-scanner subject motion with increasing symptom burden. We have therefore included state-of-the art methods for the identification and correction of outlier slices, e.g., due to subject motion or other sources of noise, which we have recently demonstrated substantially increases the temporal signal-to-noise-ratio (tSNR)^48^. Also as a limitation, TBSS is only sensitive to variability in the core parts of the major white matter pathways, and future studies targeting more peripheral parts of the white matter, e.g., closer to the cortical surface, might yield a different pattern of results. Further, conventional DTI based metrics do not allow for high neurobiological specificity^48^, e.g., due to the influence of the complexity and degree of crossing of the fibers, and further studies are needed to characterize the exact pathophysiological mechanisms.

In conclusion, we have demonstrated associations between persistent cognitive, somatic and emotional symptoms 12 months after MTBI and WM microstructural properties as measured using DTI, suggesting that DTI can reveal a biological substrate for post-concussion symptoms and global functioning in MTBI, which may have been undetected or unrecognized in the acute injury phase. Such objective evidence of injury in MTBI may play an important role in the management of patients with persisting post-concussion symptoms.

## Methods

### Ethical statement

All methods were performed in accordance with relevant guidelines and regulations. All protocols were approved by the Norwegian Regional Committee for Medical Research Ethics (REC) (2010/1899) (Oslo, Norway). All participants provided written informed consent, and all methods were carried out in accordance with the relevant guidelines and regulations of the REC.

### Subjects

Patients with acute MTBI admitted to Oslo University Hospital during a period from September 2011 to September 2013 were included in a prospective cohort study; 223 patients aged 16–65 years with recent (<24 hours) history of head trauma (hospitalization with ICD-10 diagnosis S06.0-S06.9), resulting in loss of consciousness (LOC) < 30 minutes, PTA < 24 hours and GCS between 13 and 15 were included. The lowest GCS score within the first 24 hours is reported. MTBI was defined using criteria from the American Congress of Rehabilitation Medicine^49^. Patients were categorized as having uncomplicated or complicated MTBIs according to trauma-related intracranial structural changes observed via acute CT and MRI obtained 4 weeks post-injury. Exclusion criteria were severe mental illness (schizophrenia or bipolar disorder), progressive neurologic disease, ICD-10 diagnosis of substance dependence, contraindications for MRI, and lack of Norwegian language skills.

One-hundred and sixty patients returned for the 12-month follow-up including clinical assessment and multimodal MRI, of whom 134 had available DTI data of sufficient quality. We have previously reported brain volumetric and morphometric findings based on T1-weighted MRI data from the baseline assessment in an overlapping sample^50^.

### MRI data acquisition and analysis

3 T MRI (GE Signa HDxt, GE Medical Systems, Milwaukee, WI, USA) data were obtained 12 months post-injury using two different head coils (12 channel Head/Neck/Spine (HNS) and standard 8channel GE head coil). For diffusion-weighted imaging, a 2D spin-echo whole-brain echo planar imaging pulse with the following parameters was used: repetition time (TR) = 15 s, echo time (TE) = 81.2–85.6 ms (defined as minimum), flip angle = 90°, slice thickness = 2.5 mm, field of view (FOV) = 240 × 240 mm, acquisition matrix = 96 × 96(reconstructed to128 × 128), reconstructed in-plane resolution = 1.875*1.875 mm/pix, 30 volumes with different gradient directions (b = 1000 s/mm^2^) and two b = 0 volumes with reversed phase-encode (blip up/down) were acquired. Total scan time for the diffusion MRI sequence was 8 min.

MRI data were evaluated for gross pathologies by a neuroradiologist, and a T2-weighted and T2 susceptibility-weighted angiography (SWAN) sequence were performed to depict hemorrhagic or other lesions. The 12-month MRI follow-up was performed at a median time of 530 days post-injury (inter-quartil range = 235 days), and there was no major scanner upgrade in the study period.

### DTI processing and analysis

Image analyses were done using FSL^51–53^. Processing included eddy (http://fsl.fmrib.ox.ac.uk/fsl/fslwiki/EDDY) and topup (http://fsl.fmrib.ox.ac.uk/fsl/fslwiki/TOPUP)^51,54^ to correct for geometrical distortions and eddy currents. Topup uses information from the reversed phase-encode blips, yielding image pairs with distortions in opposite directions. From these pairs, we estimated the susceptibility-induced off-resonance field and combined the two images into a single corrected one. We used eddy to detect and replace slices affected by signal loss due to bulk motion during diffusion encoding, which was performed within an integrated framework along with correction for susceptibility-induced distortions, eddy currents and subject motion^55^. Although these processing steps have been shown to strongly increase the temporal signal-to-noise ratio (tSNR)^48^ and thus largely mitigate effects of subject motion and other sources of noise on data quality, no methods for data cleaning are perfect, and possible associations between data quality and clinical features may still induce spurious clinical correlations with DTI metrics. To directly test for associations between data quality and clinical outcome, for each dataset, we calculated tSNR and maximum voxel intensity outlier count (MAXVOX)^56^. Briefly, while MAXVOX reflects intensity-related artifacts, tSNR is a proxy for global data quality. We used these quality measures both to test for associations with clinical outcome and to confirm if clinical associations with DTI remained after including tSNR in the statistical models. FA, eigenvector and eigenvalue maps were calculated using dtifit in FSL. MD was defined as the mean of all three eigenvalues, RD as the mean of the second and third eigenvalue^57^, and AD as the principal eigenvalue. Voxelwise group analysis of FA, MD, AD and RD was carried out using tract-based spatial statistics (TBSS)^58^. FA volumes were aligned to the FMRIB58_FA template using nonlinear registration (FNIRT)^59,60^. Next, mean FA were derived and thinned to create a mean FA skeleton, representing the center of tracts common across subjects. The same procedures were applied for MD, AD and RD. We thresholded and binarized the mean FA skeleton at FA > 0.2 before the resulting data were fed into voxelwise statistics.

To summarize data in a conventional neuroanatomical context we calculated mean DTI values across the skeleton and within regions of interest (ROIs) based on the intersection between the skeleton and the probabilistic JHU white-matter atlases^61,62^. In particular, for each DTI measure, we derived a set of 24 features, including 20 ROIs, 4 additional features comprising the mean value across skeleton, as well as the genu, splenium and body of the corpus callosum^63^.

### Statistical analyses

Descriptive statistical analyses were performed using SPSS for Windows, version 22 (SPSS Inc., Chicago, IL, USA). Statistical significance was reported at the 0.05 level. Descriptive statistics and PCA were also performed using Matlab (MathWorks, Inc.) and R^64^. Voxelwise non-parametric analyses were performed using Randomise^65^. Associations between the composite outcome score and FA, RD, MD and AD were tested using general linear models (GLM) while covarying for age, sex and head coil. The data were tested against an empirical null distribution generated across 5000 permutations in order to correct for multiple comparisons across space, and threshold free cluster enhancement (TFCE)^66^ was used to avoid manually defining the cluster-forming threshold. Voxel-wise maps were thresholded at p < 0.05, corrected. Average FA, MD, RD and AD within significant clusters were computed and submitted to further analysis to compute the commonly reported effect size Cohen’s *d* as follows: $d=\,\frac{2\ast t}{\surd df}$, where *t* is the t-statistic and *df* the degrees of freedom of the residuals. We tested the association between the ROI measures and the composite score using GLM, accounting for the effect of age, sex and head coil. We added DTI QC metrics (tSNR or MAXVOX, see above) to test for possible confounding effects of data quality. Correction for multiple comparisons across all ROIs tested was performed following the False Discovery Rate (FDR) procedure^67^.

### Demographic and clinical assessment

We assessed key pre-injury, acute and post-injury variables. Acute clinical data were obtained from medical records and outcomes at the 12-month follow-up.

#### Pre-injury factors

Information regarding age, sex, education level, anxiety and depression (yes/no), and employment status were obtained from the clinical interview.

#### Injury-related factors

GCS^68^ assesses the conscious state, with total scores between 3 (showing no response) and 15 (alert and well orientated). Duration of PTA was assessed in the emergency department and classified into no amnesia, less than one hour and between one and 24 hours. The presence and duration of LOC were based on medical records and classified into no LOC, < 5 min and > 5 min. Causes of injury were obtained from medical records and classified as traffic accidents, falls, violence or others.

### Outcome assessment

The main clinical outcome variable was defined as the subject weights of the primary component from a principal component analysis (PCA) across five items from three frequently used instruments assessing key aspects of recovery and function obtained 12 months post-injury:

GOSE measures global functions including independence, work, social and leisure activities and participation in social life^69^, recommended as the main outcome measure for TBI^70^. It is an 8-point ordinal scale reflecting good recovery ( > 7), moderate (5,6) and severe (3,4) disability, vegetative state (2) and death (1). The Rivermead PostConcussion Symptoms Questionnaire (RPQ) assesses frequent cognitive, emotional and somatic domains^71^. The sum of the subscores RPQ somatic, RPQ emotional and RPQ cognitive were used. Patient Health Questionnaire 9 (PHQ-9) was used to assess depressive symptoms^72^. Total score of 0–4 indicates no depression, 5–9 mild, 10–14 moderate, 15–19 moderately severe, and 20–27 severe depression, and the total score was used.

**Figure 3:**
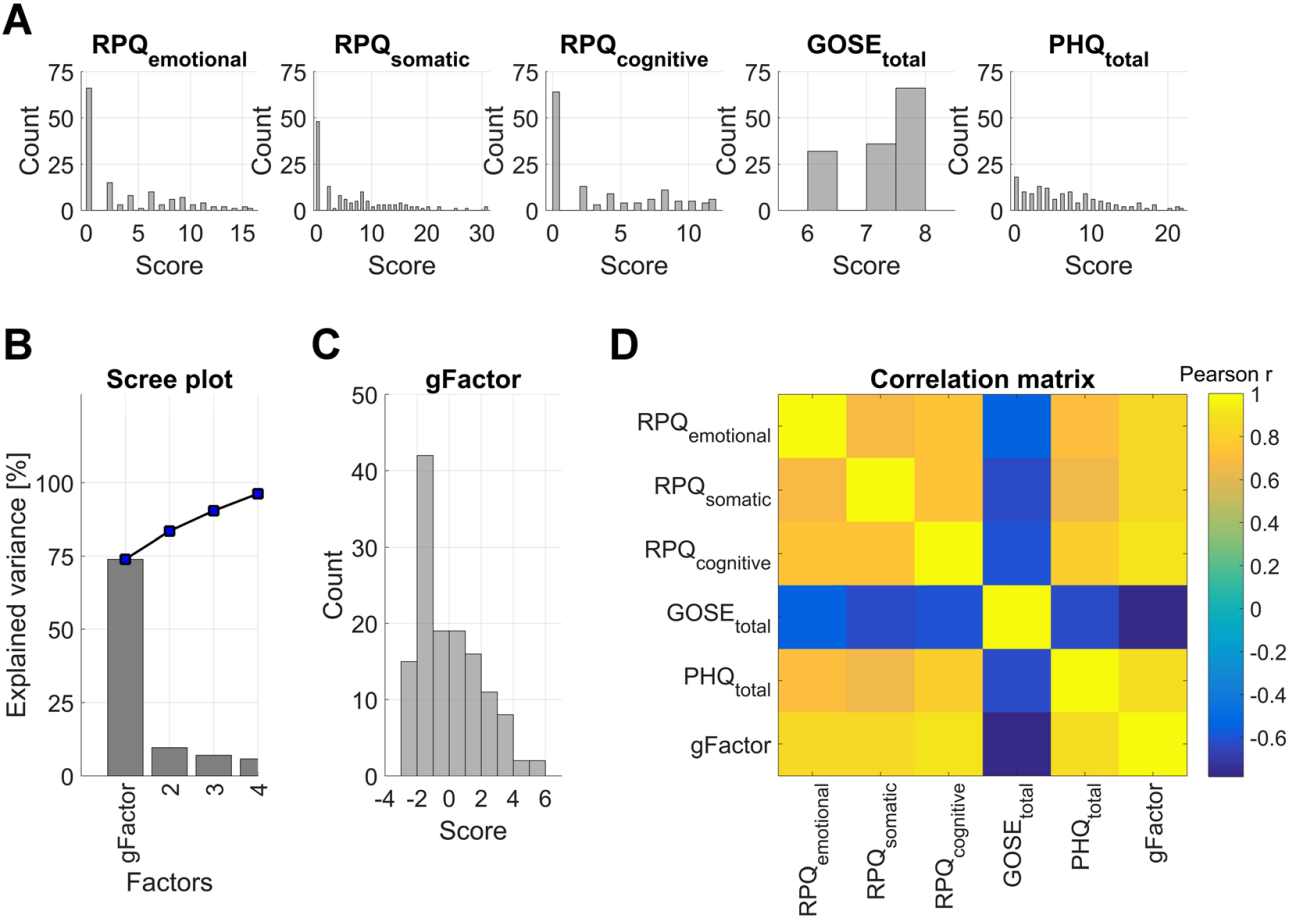
The distributions of each of the clinical raw scores (RPQ emotional, RPQ somatic, RPQ cognitive, total GOSE, total PHQ-9, panel A), the scree plot from the PCA (panel B), the distribution of the composite score (panel C), and the correlation matrix between each of the clinical variables with colors reflecting the sign and strength of the Pearson correlations (panel D).

We used the factor explaining the highest proportion of variance. This score explained 73% of the total variance in the five scales, which were well represented in this factor (coefficients; RPQ emotional/somatic/cognitive: 0.45/0.45/0.47, GOSE: 0.41, PHQ: 0.46), indicating that all outcome metrics contributed substantially to the composite score. A high composite score reflects poor outcome. Figure 3 shows the distributions of each of the clinical raw scores (panel A), the scree plot from the PCA (panel B), the distribution of the composite score (panel C), and the correlation matrix between each of the clinical variables with colors reflecting the sign and strength of the Pearson correlations (panel D).
